# MPI System with Bore Sizes of 75 mm and 100 mm Using Permanent Magnets and FMMD Technique

**DOI:** 10.3390/s24123776

**Published:** 2024-06-10

**Authors:** Jae Chan Jeong, Tae Yi Kim, Hyeon Sung Cho, Beom Su Seo, Hans Joachim Krause, Hyo Bong Hong

**Affiliations:** 1 Electronics and Telecommunications Research Institute (ETRI), 218 Gajeong-ro, Yuseong-gu, Daejeon 34129, Republic of Korea; channij80@etri.re.kr (J.C.J.); jinsun@etri.re.kr (T.Y.K.); hsc@etri.re.kr (H.S.C.);; 2 Institute of Biological Information Processing, Bioelectronics (IBI-3), Forschungszentrum Jülich, 52425 Jülich, Germany; h.-j.krause@fz-juelich.de

**Keywords:** MPI, FMMD, permanent magnet, mechanical movement, SPIO

## Abstract

We present two magnetic particle imaging (MPI) systems with bore sizes of 75 mm and 100 mm, respectively, using three-dimensionally arranged permanent magnets for excitation and frequency mixing magnetic detection (FMMD) coils for detection. A rotational and a translational stage were combined to move the field free line (FFL) and acquire the MPI signal, thereby enabling simultaneous overall translation and rotational movement. With this concept, the complex coil system used in many MPI systems, with its high energy consumption to generate the drive field, can be replaced. The characteristic signal of superparamagnetic iron oxide (SPIO) nanoparticles was generated via movement of the FFL and acquired using the FMMD coil. The positions of the stages and the occurrence of the *f*_1_ + 2*f*_2_ harmonics were mapped to reconstruct the spatial location of the SPIO. Image reconstruction was performed using Radon and inverse Radon transformations. As a result, the presented method based on mechanical movement of permanent magnets can be used to measure the MPI, even for samples as large as 100 mm. Our research could pave the way for further technological developments to make the equipment human size, which is one of the ultimate goals of MPI.

## 1. Introduction

Magnetic particle imaging (MPI) is a medical imaging technique that uses the nonlinear magnetic characteristic response of superparamagnetic iron oxide (SPIO) as a signal and field free line (FFL) or field free point (FFP) for spatial encoding. Considerable research and development has been conducted on this method since Gleich and Weizenecker published their concept and prototype in 2005 [1]. Although MPI does not provide the same anatomical information as MRI, it has attracted considerable attention owing to a few important advantages [2,3,4,5]. In terms of technology and equipment, MPI has a high resolution and a fast acquisition speed. Second, the MPI signal is proportional to the concentration of SPIO in the measurement volume; therefore, quantitative analysis is possible [6]. In terms of its use as a medical imaging device, MPI is a non-ionizing and non-radiative method; therefore, it is safer than methods using radioactive tracers, such as PET-CT [6]. Additionally, the surfaces of SPIO particles can chemically immobilize various receptors, such as antigens or antibodies [7,8]. Currently, MPI uses the characteristic response when an AC or DC magnetic field is applied to the SPIO and maps it in space. After applying a magnetic field of a certain frequency and intensity to SPIO, the applied frequency and harmonics can be obtained using Fourier transformation [9]. The current methods used to obtain the signals in MPI can be divided into two categories. The first method applies a single frequency and records higher harmonic responses [1,10], and the second method applies two frequencies and records mixed frequency responses [11,12]. In this study, the two-frequency method was adopted. The generation and intensity of these harmonics provide quantitative information on the presence of SPIO in space. When magnetic fields of two frequencies, a high-frequency component *f*_1_ and a low-frequency component *f*_2_, are applied to SPIONs, time-varying magnetization is created. When this signal is integrally transformed by a method like FFT, uneven harmonics and, additionally, sum-frequency components with an uneven number of constituents, such as *f*_1_ ± *nf*_2_ (with *n* = 2, 4, 6, … denoting an even integer), occur, in addition to excitation components *f*_1_ and *f*_2_, if there is a nonlinear magnetic object in the volume of interest [13]. Even harmonics and mixing terms consisting of an even number of constituents disappear because of the symmetry of the signal. If no nonlinear magnetic material is present, no mixed frequency terms are generated, and only excitation components *f*_1_ and *f*_2_ can be measured [13]. Note that this frequency-mixing scheme differs from the two-frequency drive field-excitation scheme used to move the FFP along the Lissajous trajectory [14]. The advantage of measuring mixing terms *f*_1_ ± *nf*_2_ instead of uneven harmonics 3*f*_1_ or 5*f*_1_ (as is performed in commercialized MPI) is that no strong high-frequency field is needed. A strong field at a low frequency *f*_2_ drives the particles close to saturation, and a weak field at a high frequency *f*_1_ is used to probe their nonlinear magnetization.

SPIO spatial information (spatial encoding) is obtained using an FFL or FFP [15]. Using an FFL or FFP implies that the magnetic field outside of the FFL or FFP is large enough for the SPIO to become magnetically saturated and will not respond to magnetic excitation [16,17]. This magnetic field can be generated using a Maxwell coil pair [1,18] or a permanent magnet [11,19]. The magnetic field created in this manner is known as the selection field. Therefore, to obtain high-resolution MPI, it is essential to create a steep gradient field. In this study, a selection field was generated using permanent magnets arranged in three dimensions. The use of permanent magnets has the advantages of a simple structure and reduced power consumption compared to the creation of a selection field using an electromagnetic coil. To create a two-dimensional or three-dimensional image from the field of view (FOV), the generated FFL or FFP must be moved. Two methods have been proposed for moving an FFL or an FFP. The first is the installation of an additional coil that creates a driving field. The method of moving an FFL or FFP using a drive field has undergone considerable research and development since Gleich and Weizenecker’s study [1]. The second is the use of a combination of a solenoid and of permanent magnets. This method was first proposed by Goodwill et al. [18]. The selection field was created using a permanent magnet, and the drive field was created by a solenoid coil. The third method involves creating a selection field using a permanent magnet and then moving it mechanically. This method replaces the role of the drive field using a motorized stage. Therefore, the method does not require power or additional devices to move the drive field. In a previous study, we presented a device that could image the spatial distribution of SPIO using permanent magnets and FMMD technology [11,19]. Another type of MPI incorporating permanent magnets mainly uses a Halbach array or has permanent magnets arranged three-dimensionally. The manufacturing method for the Halbach cylinder proposed thus far involves hardening a small magnet in epoxy resin while adjusting the angle in a circular frame, or creating a frame using nonferrous metal (mainly aluminum) to fix the positions. Although various types of MPI are being researched and developed, the development of MPI for application in humans is the ultimate goal of future research. However, until now, the maximum bore diameter is 20 cm [20], so measurement is possible only with very small laboratory animals. As mentioned earlier, the largest barrier to the expansion of an MPI system is the enormous power consumption required to create a steep gradient field and the movement of the FFL or FFP by the drive field. In this study, we present an alternative MPI system capable of measuring samples with bore diameters of up to 100 mm.

## 2. Materials and Methods

### 2.1. Instrumentation, FFL Generation Magnets and FMMD Coils

**Figure 1:**
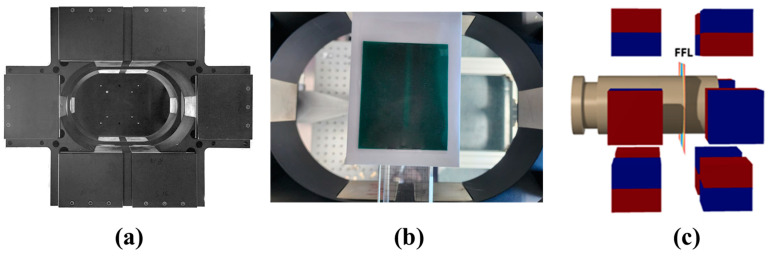
Aluminum housing (**a**) with the magnetic viewing film placed inside, showing the position of the FFL (**b**) and FMMD coil and FFL in the Halbach-type cuboid (**c**).

Rotational (RS-400-M) and translational (LS-270) stages were used to move the FFL generation magnet. A linear stage was purchased from Physik Instruments (Karlsruhe, Germany). The rotation stage and a motorized lab jack moving along the Z axis for vertical movement of the sample and coil were purchased from Namil Optical Instruments (Incheon, Republic of Korea). The travel range of the translation stage used was 305~1016 mm, and the load capacity of the rotational stage was 300 kg. Therefore, it could sufficiently handle the required travel distance of the equipment (300–200 mm) and the weight of approximately 100 kg of the FFL generation system. The FFL generation system had 24 magnets arranged in two overlapping aluminum housings, as shown in Figure 1a. All magnets used were NdFeB (N35 grade) with a size of 140 × 100 × 80 mm^3^. The average magnetic flux density measured at the surface of the magnets was 365 mT, and the standard deviation was 4 mT. The strength of the magnetic field inside the FOV was measured after mounting a Hall sensor (Sensor 2go, Infineon, Munich, Germany) on a 3-axis robot (TT tabletop robot, IAI, Shizuoka, Japan). The creation of the FFL was verified using a magnetic viewing film (CMS Magnetics, Garland, TX, USA).

The coil system for applying and measuring the excitation signals consisted of two excitation coils and a detection coil. Both the coils were wound in the same direction. The detection coil was located inside the excitation coils and had a differential structure. The bobbin of the coil system was composed of monocast nylon. Table 1 lists the parameters of the excitation and detection coils.

The two excitation signals from the function generator (BK precision 4055B) were amplified 20 times using two AC amplifiers (7224; AE Techron, Elkhart, IN, USA). The current consumed by the coil was measured using a voltage/current monitor (VMON20, AE Techron, Elkhart, IN, USA) mounted on the AC amplifier. The AC magnetic field was measured using a gauss meter with a measurement range of 10 kHz. (GM08, HIRST magnetics, Cornwall, UK). The signal measured by the detection coil was processed using a DAQ system (UML-SNAS-100; UMLogics, Daejeon, Republic of Korea). Control of the above stages, function generator, DAQ system, signal processing, and image creation was performed using software developed in-house by our laboratory based on the MFC. The control software (XIS_CONTROLLER_GUI_FOR MPI Version 1.0) was registered with the Korean Copyright Association under the number C-2019-027032. Table 2 lists the amount of current consumed and the magnetic field generated by the 75 mm and 100 mm FMMD coil systems under the experimental conditions of this study.

### 2.2. Operational Condition, Sensitivity and Spatial Resolution

To verify the operation and performance of the fabricated equipment, 70 nm SPIO purchased from Micromod (Synomag^®^, Rostock, Germany) was used. The frequencies used in the experiments were determined experimentally. After measuring the impedance of each coil, we fixed the frequency of the LF coil and changed the frequency applied to the HF coil, selecting the frequency at which the *f*_1_ + 2*f*_2_ signal was the strongest.

All experiments related to sensitivity and spatial resolution were repeated at least three times, and the results were compared with the case in which no sample was inserted. To measure the detection limit, the measurements were conducted while reducing the concentration from 25 mg/mL to 100 μg/mL (measurement volume: 100 μL). The sample was tested by placing it at the center of half of the detection coil and near the edge of the coil. To determine the spatial resolution of the system, three PCR tubes with 200 μL of SPIO solution were used. Three tubes were placed parallel to each other in the XY plane, and one more tube was placed approximately 5 mm below the Z axis. The concentration of SPIO used to measure the spatial resolution was 0.75 mg/mL (as the iron concentration), and the amount used was 100 μL.

### 2.3. Two-Dimensional and Three-Dimensional Images

The method of obtaining images in this study was back-projection based on Radon and inverse Radon. First, the magnetic system that generated the FFL moved rotationally or translationally, creating a sinogram based on the XY plane. When inverse Radon conversion was performed using the secured sinogram, the most recent FFL position was restored. To obtain a 3D image, the sample was moved along the Z axis, and this process was repeated. After moving the sample along the Z axis, 2D imaging was performed repeatedly to obtain a 3D image. The time required to obtain a single sinogram using this process was 145 s. To perform 3D MPI, samples were prepared by injecting 100 μL of Synomag-D 70 nm solution into three PCR tubes with a capacity of 200 μL. The PCR tubes were arranged in triangles at distances of 20, 40, and 30 mm; two PCR tubes were placed at the same height, and one PCR tube was placed 6 mm higher to confirm that 3D images were acquired. Sinograms were acquired eight times along the XY axis while moving the measurement sample 5 mm along the Z axis. The acquired sinogram data were subtracted from the background sinogram obtained without the sample, and 2D slice images were obtained using inverse Radon transform. To reconstruct the 2D images into 3D images, the images were visualized using the maximum intensity projection of MATLAB’s Volshow function. Sample photos were taken along the XY, XZ, and XYZ axes and co-registered with the 3D MPI images.

## 3. Results

### 3.1. Instrumentation, FFL Generation Magnets, and FMMD Coils

**Figure 2:**
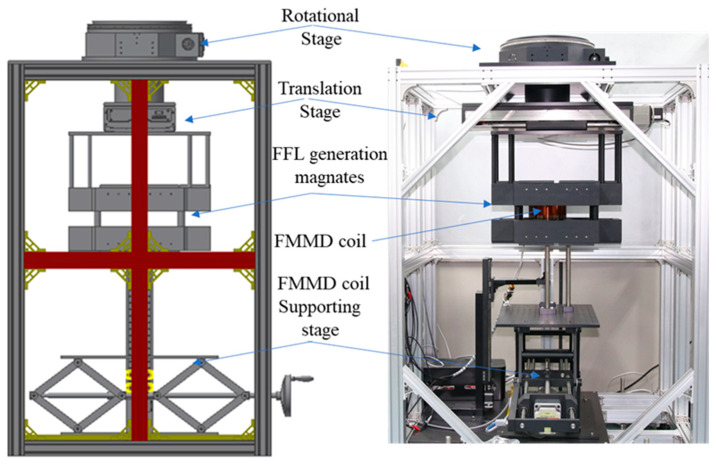
Overall diagram and photo of the MPI system.

**Figure 3:**
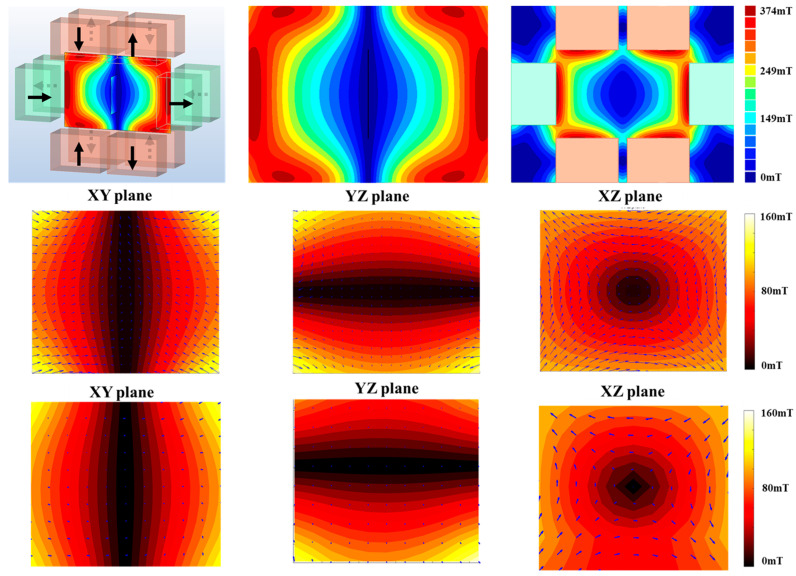
Left: magnet placement in Halbach-type cuboid, with arrows indicating magnets’ polarity (**top left**); **top middle**: simulated absolute magnetic field, showing in blue the FFL generated in the middle; **top right**: magnetic field distribution outside the cuboid. Field orientation (arrows) and distribution of absolute magnetic field strength (color) in all three orthogonal planes, simulated (**middle** row) and measured (**bottom** row).

Figure 2 shows a diagram of the MPI system and a photograph captured after implementation. It has two stages at its top that rotate and translate the magnet to generate an FFL. As mentioned previously, there is no additional module for creating a drive field in an MPI system. As shown in Figure 1 and Figure 3, the FMMD coil is located in the middle of the two magnet layers where the FFL is created. To generate the 3D MPI, the sample to be measured moves along the Z axis.

Because our research goal in this work was to generate an FFL using only permanent magnets, we investigated various arrangements of magnets in advance using simulation software (Faraday, Integrated Engineering S/W, Version 10.2, Winnipeg, MB, Canada). The drawing function of the software was used to place magnets in three-dimensional space for simulation. The type of magnet selected was “Neodym 35 MGOe Sintered”, which is similar to the magnet currently used in this experiment, and the polarity direction of each magnet was specified in the same way as in the experiment. The following structures (Figure 1a) were fabricated based on this simulation, shown in Figure 3: The results of measuring the magnetic field distribution in the sample space are in good agreement with the simulation results. All spherical objects surrounding the magnet were made of aluminum, and an anodizing treatment was performed. The bore size of the FFL generation magnet was 200 × 300 mm^2^. Therefore, there was sufficient space to move the FFL along the XY axis inside the FFMD coils with diameters of 75 and 100 mm. The strength of the internal gradient magnetic field was 2.0 T/m, and Figure 1 shows that the FFL was generated along the X and Y axes. The magnet shape proposed by other research teams is such that many small magnets are solidified with epoxy resin, or a Halbach cuboid shape is created by adjusting the angle of the square magnet. This form is typical according to Halbach’s theory. However, the following issues need to be addressed in the experimental realization, especially with regard to subsequent commercialization. Even if the magnets have only slight differences in the strength of their magnetic fields, and even if a theoretical calculation with these varying magnet strengths is performed, the measurement results after the resin has hardened often differ from the calculation. In reality, determining the relative and absolute angles for each magnet is difficult. However, we were able to alleviate the problem of controlling the magnets’ angles and positions by orienting all magnets either vertically or horizontally in our simulation.

In the case of the currently proposed system, the FOV in the XY two-dimensional plane is 120 × 120 × π/4 mm^2^, and in the case of the Z axis, it depends on the working distance of the stage that moves the sample up and down. Because the current distance that can be moved up and down is 100 mm, the size of the sample that can be measured simultaneously is (120 × 120 × π/4) × 100 mm^3^.

### 3.2. Sensitivity

The results of the sensitivity experiments using Synomag 70 are listed in Table 3. The total solid content of the stock solution of Synomag 70 used in this study was 25.0 mg/mL, with an iron of approximately 50%. Additionally, in this experiment, it was diluted up to 250 times, so the iron-based concentration was approximately 125–12,500 µg/mL. As the amount used for the measurement was 100 μL, the amount detected was 12.5–1250 μg. To confirm the detection limit, we performed a paired-sample t-test using the control data and 0.125 mg/mL data. The results show that the population mean of 0.125 mg/mL at the 0.05 level was significantly different from that of the control group. Therefore, it is shown that both the 75 and 100 mm coils used in this paper could measure Synomag 70 below 0.125 mg/mL (absolute amount: 12.5 µg) regardless of location. However, the measurement results of the two coils at a high concentration showed a signal level about 3.5~3.9 times higher when measuring with the 100 mm coil compared to using the 75 mm coil. At lower concentrations, this figure appears to be reversed, but comparison to the control still shows that the 75 mm coil could measure the SPIO at lower concentrations. The difference between the signals at the center and edge of each coil was significant. When using the 75 mm coil in the experiment, there was an almost twofold difference at the highest concentration and no significant difference at the lowest concentration. However, there was an approximately three-fold difference in the control measurements. A similar situation was measured using the 100 mm coil. At the highest concentration, a signal difference of about three times was measured, whereas at the lowest concentration, there was almost no difference.

### 3.3. Spatial Resolution

**Figure 4:**
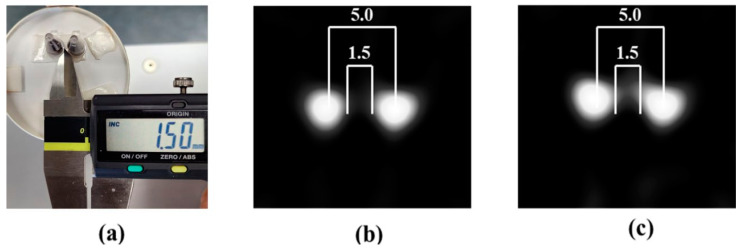
Two-dimensional (XY plane) spatial resolution of the MPI presented in this work. (**a**) Photograph of the samples used, with distance measured in mm. (**b**) MPI obtained using a 75 mm coil, and (**c**) MPI using a 100 mm coil.

In this experiment, a 0.2 mL PCR tube was used as a container. Because the detection limits in the XY plane were different, the measurements were performed at the center and outer corner of the FMMD coil (Table 3). Under the test conditions used in this work, separated images could be obtained if the distance between the two samples was 1.5 mm or more. And when the center distance between the two samples was 5.0 mm, separated images could be obtained (Figure 4). Even when measured in the center, if the concentration of the sample was sufficient, there was a difference in the intensity of the measurement signal at each measurement point, but this did not affect the resolution. However, when the sample concentration was low (0.39 mg/mL), it was often difficult to distinguish the signal from the resolution.

### 3.4. Three-Dimensional MPI Image Using Phantom Sample

**Figure 5:**
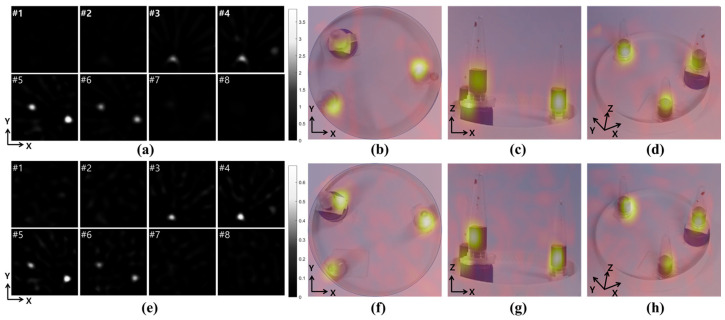
Three-dimensional MPI was reconstructed using 75 mm coils (**a**–**d**) and 100 mm coils (**e**–**h**). The black and white images at the left show 2D slice images taken at 5 mm increments along the Z axis, with #1 corresponding to Z = 0 mm to #8 at Z = 35 mm. The colored images at the right show sample photos overlaid with 3D MPI images along the XY plane (**b**,**f**) and the XZ plane (**c**,**g**), and tilted to show the XYZ axes (**d**,**h**).

Sample photos were taken along the XY, XZ, and XYZ axes and co-registered with the 3D MPI images. As shown in Figure 5a,e, imaging was performed while moving the sample in 5 mm increments along the Z axis, so the sample at a high position was first identified in the 2D image, and then three points and two points were shown in the 3D image. As verified in the detection limit experiment, it was confirmed that the phantom samples were imaged with stronger signals in the 75 mm coil than in the 100 mm coil. In (b) and (f), it is confirmed that the sample photographs and imaging results match the XY axis, which is the top view of the sample. When the sample was viewed from the sides, as shown in (c) and (g), it was confirmed that the images were co-registered at different positions depending on the height of the sample. Finally, as shown in (d) and (h), it was confirmed that the positions of the samples matched from a 3D perspective.

## 4. Discussion

MPI is one of the most recently developed preclinical imaging techniques compared to the well-established methods such as MRI, X-ray, ultrasound, and PET. Since the concept and prototype of the equipment were announced in the early 2000s, equipment for analyzing small animals and samples has been commercialized [21,22]. However, to apply it to humans, technical and scientific issues, such as understanding the impact of strong magnetic fields on humans, developing biocompatible SPIO, and enlarging the equipment, must be resolved [20,23]. Among these, the development of equipment with a bore size large enough for a person to enter (regular: 50–60 cm, wide: 80 cm, based on MRI) is a very important issue [24], as it must be used in preclinical experiments and tests, such as the development and safety confirmation of SPIO for humans. Based on the papers and presentations published to date, it has been reported that the usual bore sizes of MPI range from 3 cm to 20 cm [1,14,20], and thus far, MPI at a level suitable for small animals is common [25]. To maintain or increase the resolution as the size increases, a narrowly-shaped FFL must be formed; therefore, a very high gradient strength is required. This requires additional elements, such as cooling systems and a Tx/Rx signal chain capable of handling high voltages and currents, which presents a significant obstacle to the large-scale dissemination of MPI. Among the MPI systems investigated to date, the equipment that can image the largest sample uses a yoke-type electromagnet and a robot system [23]. The MPI proposed in this study reduces the complexity of manufacturing and operating the drive field used in the existing research and replaces the selection field with a permanent magnet. As our current equipment has bore sizes of 75 and 100 mm, we were able to distinguish samples spaced at 1.5 mm intervals (center to center: 5.0 mm). The proposed FFL generation system uses an array of permanent magnets to generate a selection field and does not require the creation of a separate drive field; therefore, the overall power requirement can be kept below 500 W, reducing image acquisition time and additional resources.

In this study, an FMMD-based multilayered solenoid coil constituting three layers was located at the center of the detection coil (capable of capturing the strongest signal and maximizing the excess of the differential coil). Therefore, the sample size could be less than half of the bore size of the FFL generation magnet responsible for generating the selection field. Based on the FFL generation system, even samples approximately 150 mm in size can be measured. However, when the length exceeds 120 mm, the signal level of the *f*_1_ + 2*f*_2_ harmonics is too low to allow imaging. Therefore, in this study, we experimented with coils with bore sizes of 75 and 100 mm. As demonstrated in other studies with different detection systems, the difference in sensitivity at the center of the coil and at the edge of the coil was found to be significant for both the 75 and 100 mm coils. The signal became stronger toward the edges and weaker toward the center. This indicates that the strength of the signal reaching the coil varies depending on where the signal is generated. Because this problem varies depending on the size of the coil and the characteristics of the tracer used, additional research will be needed in the future. In this paper, we propose MPI technology for samples with diameters of 75 and 100 mm, which are quite large for the current MPI technology. Contrary to other existing research based on electromagnets or solenoid coils, this study attempted to increase the size of the equipment based on a three-dimensional arrangement of permanent magnets. As a result of these attempts, it was possible to build an MPI system with a resolution of less than 5 mm while using sample measurement sizes of 75 and 100 mm.

To expand the measurement range (FOV) of the system, a powerful and large permanent magnet is required; however, larger permanent magnets exhibit more relative variation in their moments than smaller magnets, affecting the resolution and the generation of artifacts. In addition, the precise control of the mechanical system and the increase in measurement time due to the increase in the weight of the magnet are technical challenges that must be solved. Just as various types of MRI and X-rays have been developed according to need, we believe that MPI will follow in these footsteps. Although technologies can be applied in fields where securing high-resolution and high-speed images is important, there may be a need for MPI equipment that is efficient in manufacturing and operation, even if the technical requirements are low. The approach proposed in this study demonstrates the feasibility of producing an MPI system that can be used in such fields.

## 5. Conclusions and Outlook

In this study, MPI equipment with bore sizes of 75 mm and 100 mm was developed by combining a three-dimensional array of permanent magnets with mechanical movement and FMMD technology, making it possible to image the distribution of SPIOs in three-dimensional space. Although the MPI system proposed in this study has a relatively large bore size compared to existing MPI systems, it consumes very low power compared to electromagnet-based MPI, due to the mechanical movement of the permanent magnets. The method suggested in this study is expected to foster large-sample MPI technology, which is currently emerging as a new methodology in pre-clinical imaging. Despite the positive potential for the development of MPI technology, there are technical issues that need to be further addressed. The use of large research magnets simplifies design and manufacturing, but due to their large variation in magnetic moments, the image resolution is impaired. The accuracy of the mechanical movement of the magnets can have a significant impact on the resolution, and the time required to acquire images is longer than that of MPI systems using electromagnets for field generation. Therefore, it is deemed necessary to develop a shimming technology for permanent magnet-based MPI, realize improvements in mechanical movement, and devise algorithms for correcting imaging errors.

## Figures and Tables

**Table 1:** Parameter of the FMMD coils of the systems with 75 mm and 100 mm bore sizes, respectively. Det.—detection; HF—high-frequency; LF—low-frequency.

**75 mm**	**LF Coil**	**HF Coil**	**Det. Coil**
Width (mm)	116.5	116.5	40
Inner radius (mm)	47.3	46.5	37.5
Height (mm)	4	0.8	1.8
Wire diameter (mm)	0.4	0.4	0.3
No. of layers	10	2	6
No. of windings	233	223	126
**100 mm**	**LF Coil**	**HF Coil**	**Det. Coil**
Width (mm)	116.5	116.5	40
Inner radius (mm)	62.6	61	50
Height (mm)	4	0.8	1.8
Wire diameter (mm)	0.4	0.4	0.3
No. of layers	10	2	6
No. of windings	233	223	126

**Table 2:** The current consumed and the magnetic field strength generated by the 75 mm coil and 100 mm coil. All LFs were based on 50 Hz operation. The 75 mm HF coil operated at 4.232 kHz, and the 100 mm HF coil operated at 4.695 kHz.

	Current (mA)		Magnetic Field (mT)	
	LF	HF	LF	HF
75 mm	56.18	80.34	7.15	0.64
100 mm	37.16	66.38	4.41	0.33

**Table 3:** Measurement of signal changes depending on the amount of sample at the edge and center of 70 mm and 100 mm FMMD coils. For each SPIO concentration, the average signal and its standard deviation are given for the sample positions at the edge and the center of the coils.

**75 mm**											
Position	Conc. (mg/mL)	25.00	12.50	6.25	3.13	1.56	0.78	0.39	0.25	0.12	0
Edge	Average (µV)	3948.1	1765.4	916.6	501.3	290.2	180.1	131.0	106.5	95.1	86.2
	STDEV (µV)	210.0	19.7	14.1	2.4	2.9	3.0	1.8	2.6	1.9	1.9
Center	Average (µV)	2006.5	917.2	500.2	291.0	189.0	132.8	108.4	96.3	90.5	29.4
	STDEV (µV)	87.9	8.5	5.2	2.1	2.4	2.3	2.2	2.0	1.9	49.2
**100 mm**											
Position	Conc. (mg/mL)	25.00	12.50	6.25	3.13	1.56	0.78	0.39	0.20	0.10	0
Edge	Average (µV)	1338.1	695.5	406.8	277.2	206.2	169.2	152.0	143.1	138.9	135.5
	STDEV	16.7	13.9	15.7	7.6	7.1	6.3	6.0	5.4	5.0	4.6
Center	Average (µV)	562.4	330.3	234.7	186.1	161.3	148.2	141.7	138.2	136.6	135.2
	STDEV	7.1	9.9	7.7	6.3	6.2	6.0	5.7	5.3	4.5	4.5

## Data Availability

Data are contained within the article.
